# Wallop of Symptoms and Co-morbidities on COVID-19 Outcome

**DOI:** 10.2174/1874306402115010046

**Published:** 2021-11-10

**Authors:** Heba H. Abo ElNaga, Hesham A. AbdelHalim, Mohamed Abdellatif, Haroun BG, Basem Elnagdy, Taghreed Ashraf, Bahaa ElNaggar, Passant S. Eldin, Ismail TA, Beshoy Mosaad, Tasbeeha Ismail, Rasmy Boules, Shawky Methuselah, Paula Rafaat

**Affiliations:** 1 Pulmonary Medicine Department, Faculty of Medicine, October 6 University, Giza, Egypt; 2 Pulmonary Medicine Department, Faculty of Medicine, Ain Shams University, Cairo, Egypt

**Keywords:** COVID 19, Symptoms, Co-morbidities, Outcome, Wallop, Questionnaire

## Abstract

**Background:**

Fever, cough, fatigue, and myalgia are usually the original clinical picture of the COVID-19 pandemic, which appears non-specific and not exclusive.

**Objectives:**

To illustrate the clinical picture pattern and assess the prevalence of underlying co-morbidities and their correlation with the severity of COVID-19 infected patients.

**Methods:**

A cross-sectional online survey included 580 participants who were either suspected or confirmed with COVID-19 infection.

**Results:**

The severity of the disease significantly correlates with both age (p=.01) and the time lag of the diagnosis of COVID-19 (p=.03). Hypertension (p=.015) and diabetes mellitus (p<.01) were significantly associated with the duration of symptoms. A wide range of ages (21-60 years) seemed to be the only risk factor for the severity. When symptoms were tested, dyspnea appeared to be the most prevalent symptom, predicting a more severe disease (OR= .066, 95% CI: .022- .200), followed by diarrhea (OR= .285, 95% CI: .122-.663), then fever (OR= .339, 95% CI: .139-.824). During the examination of co-morbidities influences on the severity, the only major co-morbidity that predicted a more severe disease was IHD (OR= .218, 95% CI: .073- .648), p= .006.

**Conclusion:**

Special consideration is required for patients with COVID-19 with an associated longer gap between symptoms and diagnosis and associated co-morbidities including hypertension, diabetes, and established chronic kidney disease (CKD), for which this study proved its profound influence on the severity of the illness and duration of symptoms.

## 1 INTRODUCTION

The clinical picture of the COVID-19 pandemic reveals that most patients (81%) had minor symptoms, an influenza-like sickness, or mild pneumonia, and 19% had severe or life-threatening pneumonia [1]. Clinical descriptions indicate that fever, cough, fatigue, and myalgia are usually the foremost symptoms; the manifestation of COVID-19 appears non-specific, and only symptoms cannot direct to suppose a case without discussion [2-4].

Previous studies reported the clinical features of patients with underlying diseases, including cardiovascular disease, diabetes, hypertension, and chronic obstructive pulmonary disease. Subsequently, the patients admitted to the Intensive Care Unit (ICU) had a higher number of co-morbidities than those not admitted to the ICU [2, 5].

Therefore, understanding and outlining the relationship between chronic diseases and severe COVID-19 patients would assist the healthcare providers in helping vulnerable populations and assessing the risk of complications. Another point is that the symptoms’ profile and its relationship to the severity of the infection are essential in planning a feasible approach to detect cases and assess the pattern of the disease rapidly. This will improve treatment outcomes and death rates [6].

Assessing the relationship between symptom severity and co-morbidities associated with COVID-19, outcomes may postulate some comprehensions of the disease and its risk factors.

This study aims to use data from a cohort of suspected patients and real-time COVID-19- confirmed PCR to illustrate the pattern of symptoms and assess the prevalence of underlying co-morbidities and its correlation with the severity of COVID-19 infected patients.

## 2 PARTICIPANTS AND METHODS

### 2.1 Study Design and Participants

Following the Egyptian Government guidelines (to the public) for reducing face-to-face communication and home isolation, we used an online snowball sampling procedure to collect data from Egyptian residents aged ≥ 18 years old during the period between 16^th^ January and 30^th^ June 2020.

We designed an online questionnaire using Google forms, then shared the link of the questionnaire with numerous Facebook groups hosting netizens from the four governorates. Facebook is the most prevalent social network among Egyptian netizens [7]. We asked respondents to forward the questionnaire to their eligible friends on their social network contact lists.

After reviewing previous studies, we determined the following criteria to calculate the minimum sample size: population size of 999,999, expected frequency of 50%, confidence level of 95%, and a margin of error of 5%. However, we then sent the survey to more than twice the minimum sample size to overcome any unexpected invalid questionnaires. Therefore, the calculated lowest sample size comprised of 384 patients. A total of 650 patients were enrolled in the study. Seventy questionnaires were excluded from the respondents, 20 reported younger than 18 years, and the remaining 50 were either taken by Egyptian expatriates (2 respondents) or were incomplete questionnaires (48 respondents).

We calculated the minimum sample size using the Epi-Info version 7 StatCalc.

The participants included were either suspected or confirmed cases of COVID-19 infection.

Suspected COVID-19 infection was defined as acute onset of ≥ 3 of the following symptoms: fever, cough, sore throat, coryza, general weakness/fatigue, headache, myalgia, dyspnea, anorexia/nausea/vomiting, diarrhea, altered mental status. Or patients with severe acute respiratory illness (SARI): acute respiratory infection with history of fever or measured fever ≥ 38°C and a cough; onset within last ten days; requiring hospitalization with radiological and/or laboratory findings.

A confirmed COVID-19 infection was defined as a person with laboratory confirmation (Molecular testing (PCR) with a nasopharyngeal swab) of COVID-19 infection, irrespective of clinical signs and symptoms [8].

Mild disease: Symptomatic patients meeting the case definition for COVID-19 without evidence of viral pneumonia or hypoxia.

Moderate disease (pneumonia): Adolescent or adult with clinical signs of pneumonia (fever, cough, dyspnea, fast breathing) but no signs of severe pneumonia, including SpO_2_ ≥ 90% on room air.

Severe disease (Severe pneumonia): Adolescent or adult with clinical signs of pneumonia (fever, cough, dyspnea, fast breathing) plus one of the following: respiratory rate > 30 breaths/min, severe respiratory distress, or SpO_2_ < 90% on room air [9].

### 2.2 Data Collection

The survey was designed as an anonymized online questionnaire of 13 questions. The questionnaire had four components: the demographic data of the respondents, the clinical presentation and the duration of illness, co-morbidities, and diagnostic method and site of management.

We used a self-selected online survey method of nonprobability sampling to recruit participants via social network posts (mainly WhatsApp and Facebook), asking the Egyptian population’s age (18 years or older) to answer the survey. This sampling method is particularly suitable in a confinement situation where the mobility and social contact of the population are significantly reduced. Thus, the online distribution of the survey enabled fast access to it by a large number of people.

The questionnaire was piloted on 25 random people to ensure the validity and practicability of the questions. The questions were written in Arabic. Once all the bugs were fixed, and minor feedback about the wording of the questions was addressed, the author proceeded to deploy the survey widely.

Subjects younger than 18 years old and older than 90 years old were excluded from the study.

The study was approved by the official ethical committee of the Faculty of Medicine, October 6 University, an accredited institution by the Ministry of Higher Education of Egypt, located in Giza governorate, as a general clearance to the chest department for further COVID-19 related studies.

### 2.3 Data Analysis

Association between data variables was done using Chi-square (χ2) test for categorical variables. Simple correlations between variables were tested using Pearson’s correlation. A logistic regression test was performed to explore the concurrent influences of different variables on the severity of COVID-19 illness. Statistical analyses were done using the statistical package for the social sciences (SPSS version 17; SPSS Inc., Chicago, Illinois, USA) statistical software. Significance was considered at a P value less than 0.05.

## 3 RESULTS

### 3.1 Demographic Data

Six hundred and fifty subjects with either suspected or confirmed cases of COVID-19 respiratory infection were involved in the study. Forty-eight patients did not complete the questionnaire, 22 patients were excluded (20 patients were less than 18 years old, and two were non-Egyptians), which amounted to 580 participants who completed the study. The patients were specifically from six Egyptian governorates: Cairo (184), Giza (105), and Alexandria (112) in northern Egypt and Beni-Suef (36), Fayoum (81), and Assiut (62) in southern Egypt. We randomly selected them for data collection. Cairo is the capital of Egypt and the most populous governorate in the country, whereas Assiut is the most crowded governorate in southern Egypt. Beni-Suef and Fayoum are two of the most underprivileged governorates with significant deficits in health and educational services. In contrast, Alexandria represents the country’s modern face with better health and educational services [10].

Characteristic data, including demographic data, method of diagnosis, site of management, the duration of symptoms, and co-morbidities, together with their correlation with the disease severity, showed a significant correlation of the disease severity with age, presence of IHD, HTN, and D.M. (Table **1**).

### 3.2 Correlations with COVID-19 Illness Duration

Deterioration of COVID-19-related illness appeared to progress with older age as well as in subjects having tested all co-morbidities (IHD, HTN, D.M., CKD) except chronic respiratory diseases.

Additionally, there was a significant correlation between the disease severity and the time lag between the onset of symptoms and the diagnosis of COVID-19.

Considering the relationship between co-morbidities and the duration of symptoms, subjects with hypertension or diabetes mellitus have a significantly more extended period of symptoms than others. 21.3% of the hypertensive patients and 17.4% of the diabetic patients had more than 21 days of illness compared to chronic respiratory disease (13.5%) and CKD (11.1%). Moreover, IHD patients with longer durations of illness were substantial (23.1%), but IHD was not statistically significantwith the duration of illness (Table **2**).

### 3.3 Logistic Regression Models

A logistic regression test was used to predict the influence of demographic data variables on the disease severity (Table **3**).

Overall, the logistic regression model explains 11.9% of the disease severity (the overall accuracy of this model is 93.6%, with a predicted probability of 0.5 or greater).

Some age groups appeared to be the principal predictor for the severity (odds ratio (OR) = .476, 95% confidence interval (CI): .066– 4.037, P<0.001) but interestingly, a wide range of age clusters (from 21 to 60 years) was significant as a risk factor for the severity.

A new logistic regression model was created when symptoms were tested as predictors for the severity of the disease (Table **4**).

Overall, 28.8% of the severity is explained by this model, with an overall accuracy of 93.6% (with a predicted probability of ≥ 0.5). Dyspnea appeared to be the most accurate indicator symptom in predicting a more severe disease (OR= .066, 95% CI: .022- .200), followed by diarrhea (OR= .285, 95% CI: .122-.663), then fever (OR= .339, 95% CI: .139-.824).

We created another logistic regression model to predict co-morbidities as risk factors for the disease severity. IHD was the only significant comorbidity that predicted a more severe illness (OR= .218, 95% CI: .073- .648), p= .006. Overall, this logistic regression model explains 10.6% of the disease severity (the overall accuracy of this model is 93.6% with a predicted probability of 0.5 or greater).

## 4 DISCUSSION

This study reports a trend of presented symptoms and severity in 600 COVID-19-infected participants (455 suspected and 145 confirmed subjects).

The study revealed that patients aged from 21 to 60 years are more susceptible to severe illness and more likely to be admitted to the hospital and ICU. According to other studies, this could be due to the alterations in lung anatomy and muscle atrophy, which results in changes in physiologic function, a decrease of lung reserve, reduction of airway clearance, and deficient defense barrier function [11].

Moreover, when a logistic regression test was used to determine the influence of demographic data variables on the disease severity, the age of the participants was the most important predictor.

Interpretation of the relationship between co-morbidities and the duration of symptoms revealed that subjects with associated hypertension or diabetes mellitus have a significantly more extended period of symptoms than others. Many studies worldwide concluded this observation [12-15].

It is essential to note that the lag between the onset of symptoms and the medical diagnosis is a crucial factor to reflect on the prognosis of COVID-19 infection. It was evident in this study by the significant correlation between the gap flanked by the onset of symptoms and diagnosis of the illness and the disease severity.

In contrast to those with chronic respiratory diseases, this co-morbidity showed an insignificant correlation with the severity of COVID infection. Additionally, smoking did not significantly impact the severity of the disease, which opposed the results of previous studies that had verified that smoking enhances the risk of progressing and severe illness in COVID-19 [16-19].

This was rationalized by many hypotheses based on the fact that a low reported prevalence of chronic respiratory diseases such as asthma and COPD in patients diagnosed with COVID-19, might be due to one or several factors: such as considerable underdiagnosis or poor recognition of the chronic respiratory disease in patients [20]. Moreover, many studies show that inhaled corticosteroids (alone or combined with bronchodilators) inhibit coronavirus replication and cytokine production [21, 22].

Vardavas *et al*. [23] analyzed data from 5 studies totaling 1549 patients and calculated a relative risk that indicated an insignificant relationship between smoking and the severity of COVID-19.

There are currently no peer-reviewed studies that directly estimate the risk of hospitalization with COVID-19 among smokers [24].

On the other hand, chronic renal disease as a co-morbidity played a significant role in prolonging symptoms, confirming other authors’ testimonies in a previous study [25].

Therefore, regarding the distinction of SARS-CoV-2 infection compared with other respiratory viral infections, patients presenting with severe illness and a longer duration of symptoms displayed more cardio-metabolic co-morbidities than other chronic respiratory diseases.

The study of Xiao *et al*. included hypertension in the risk score but neither obesity nor diabetes [1], which were previously strongly associated with poor outcomes in patients with COVID-19 [15].

Noticeably, a considerable ratio of patients – initially presented with dyspnea, diarrhea, and fever – build up an increased risk of developing severe disease. Those patients would benefit from early and more aggressive intervention. We proved it by a new logistic regression model while investigating symptoms as risk factors for disease severity.

Moreover, the authors applied another logistic regression model, which revealed that the associated co-morbidity of ischemic heart disease showed a substantial influence on the severity of the disease. At the same time, a previously published article found that hypertension is a more influencing co-morbidity [26].

## CONCLUSION

Patients with COVID-19 who have a longer time lag between the onset of symptoms and diagnosis require special consideration. Likewise, the associated co-morbidities, including hypertension, diabetes, and established chronic kidney disease, proved to profoundly influence the severity of the disease and the persistence of symptoms.

## Figures and Tables

**Table 1:** Data description and correlation with COVID-19 severity.

	N=580	p-value
**Age (years)**		
18-20	150 (25%)	< .01*
21-30	155 (25.8%)	
31-40	139 (23.2%)	
41-50	79 (13.2%)	
51-60	35 (5.8%)	
61-70	17 (2.8%)	
70+	5 (.8%)	
**Sex**		
Female	366 (63.1%)	0.25
Male	214 (36.9%)	
BMI (kg/height^2^)	30.41± 18.44	0.49
**Smoking**		
Non-smokers	493 (85%)	0.51
Smokers	87 (15%)	
**Method of Diagnosis**		
Clinical	296 (51.03%)	<.01*
Internet	143 (24.66%)	
PCR positive	141 (24.31%)	
Onset to diagnosis gap (days)	8.5	0.03*
**Site of treatment**		
Home	543 (93.6%)	< .01*
Hospital	28 (4.8%)	
ICU	9 (1.6%)	
**Co-morbidity**		
Chronic respiratory	37 (6.4%)	0.06
IHD	24 (4.1%)	< .01*
CKD	9 (1.6%)	.04*
HTN	75 (12.9%)	< .01*
DM	46 (7.9%)	< .01*

Data are presented as mean±SD for quantitative data or frequency (%) for categorical data. IHD, ischemic heart disease; CKD, chronic kidney disease; HTN, hypertension; D.M., diabetes Mellitus. *P<0.05, statistically significant.

**Table 2:** Association between co-morbidity and duration of the disease.

	Duration (Days)			*X^2^*	*p*
	7-14	15-21	21+		
Chronic respiratory	22(59.5%)	10(27.0%)	5(13.5%)	0.630	0.730
IHD	15 (57.7%)	5 (19.2%)	6 (23.1%)	4.431	0.109
CKD	6(66.7%)	2(22.2%)	1(11.1%)	0.019	0.990
HTN	37 (49.3%)	22 (29.3%)	16 (21.3%)	13.387	0.001*
DM	21 (45.7%)	17 (37.0%)	8 (17.4%)	8.396 patients	0.015*

Data are presented as frequency (percentage); IHD, Ischemic Heart Disease; CKD, Chronic Kidney Disease; HTN, Hypertension; D.M., Diabetes Mellitus; X^2^, Chi-Square value. *P<0.05, statistically significant.

**Table 3:** Logistic regression for COVID-19 severity concerning the subject demographic data.

	OR (95% CI)	*p*
**Age (Years)**		
*18-20*	0.043 (.005-.506)	0.998
*21-30*	0.067 (.009-.749)	0.003*
*31-40*	0.103 (.014-.906)	0.009*
*41-50*	0.115 (.015-3.426)	0.025*
*51-60*	0.476 (.066-4.037)	0.040*
*61-70*	0.484 (.058-1.954)	0.461
*70+*	0.000 (.421-4.416)	0.503
Male gender	0.907 (.440-1.021)	0.803
Current smokers	1.395 (.973-.342)	0.572
BMI (kg/height^2^)	0.997 (.000-.506)	0.801

Data are presented as odds ratio (95% CI), OR odds ratio; CI, confidence interval. *P<0.05, statistically significant.

**Table 4:** Logistic regression for COVID-19 severity concerning the subject symptoms.

-	OR (95% CI)	*p*
Insomnia	(1.847).829-4.111	0.133
Diarrhea	0.285(122-.663)	0.004*
Vomiting	(1.344).564-3.200	0.505
Myalgia	(1.467).654-3.286	0.352
Ageusia	(1.126).367-4.025	0.749
Anosmia	(3.028).929-9.872	0.066
Cough	(0.907).421-1.966	0.803
Dyspnea	0.066(.022- .200)	< .01*
Fever	0.339(139-.824)	0.017*

Data are presented as odds ratio (95% CI), OR odds ratio; CI, confidence interval. *P<0.05, statistically significant.

## Data Availability

The data supporting the findings of the article is available in the [Zenodo Repository] at https://zenodo.org/badge/DOI/10.5281/zenodo.5543697.svg, DOI [10.5281/zenodo.5543697].

## LIST OF ABBREVIATIONS

COVID-19: = Corona Virus Disease-2019
RT-PCR: = Real Time-Polymerase Chain Reaction.
IHD: = Ischemic Heart Disease
CKD: = Chronic Kidney Disease
HTN: = Hypertension
DM: = Diabetes Mellitus.
